# Histopathological Changes of Placenta Associated with Maternal Anaemia in Northeast Ethiopia: A Comparative Study

**DOI:** 10.4314/ejhs.v30i5.18

**Published:** 2020-09

**Authors:** Teshome Gebremeskel, Abay Mulu, Solomon Kumbi, Wondwossen Ergete

**Affiliations:** 1 Department of Anatomy, College of Health Sciences, Woldia University, Amhara Ethiopia: teshomefirst12@gmail.com; 2 Department of Anatomy, College of Health Sciences, Addis Ababa University, Addis Ababa, Ethiopia: abaymulu@gmail.com; 3 Département of Obstetrics and Gynecology, College of Health Sciences, Addis Ababa University, Addis Ababa, Ethiopia: solkumbi@gmail.com; Département of Pathology, College of Health Sciences, Addis Ababa University, Addis Ababa, Ethiopia, wondwossen_ergete@yahoo.com

**Keywords:** Anaemia, Placenta, Histopathology, Ethiopia

## Abstract

**Background:**

Anaemia during pregnancy affects about half of all pregnant mothers in developing countries; it is the major causes of indirect maternal mortality. Anaemia can directly cause poor growth of fetus in utero due to inadequate oxygen flow to the placental tissue or it is indirect indicator of maternal nutritional deficiency. Mal-development of placenta is the leading cause of maternal and perinatal mortality and an important factor of fetal growth retardation. The aim of this study was to compare histopathological changes of placenta associated with maternal anaemia.

**Methods:**

A comparative cross-sectional study was conducted from May-June, 2018 in Dessie Referral Hospital. A total of 66 placentas (33 anaemic and 33 non-anaemic) were collected after delivery. EPI data version 4.2.0 was used to enter the data while the data were analyzed by using SPSS version 22. Chi-square and one-way ANOVA were used to analyze the data.

**Results:**

In pregnancies with maternal anaemia, 75.7% of anaemic placentas terminal villi vessels were increased in number, compared to 15.1% in non-anaemic (p=0.001). Placental calcification was 72.7% in anaemic groups compared to 54% in non-anaemic groups. However, it was insignificant (p=0.12). Intervillous space was wider in anaemic compared to non-anaemic groups (p<0.001).

**Conclusions:**

Chorionic villi capillaries were increased in number, and it was dilated in anaemic placenta, compared to non-anaemic. Intervillous space was significantly wider in anaemic placenta.

## Introduction

Anaemia during pregnancy affects about half of all pregnant mothers in developing countries and it is the major cause of indirect maternal mortality; i.e., severe anaemia contributes to the risk of maternal death in cases of hemorrhage (1). Anaemia can directly cause poor growth of fetus in utero due to inadequate oxygen flow to the placental tissue, or it is indirect indicator of maternal nutritional deficiency. Globally, anaemia affects around 32.4 million (38.2%) of pregnant women. It is a severe public health problem in Southeast Asia (48.7%) and Africa (46.3%). It is estimated that anaemia is responsible for 20% of all maternal deaths in sub-Saharan Africa (2–4).

The prevalence of anaemia among pregnant women in Brazil was reported as 53.7% (5). In Ethiopia, anaemia prevalence among women aged 15–49 years declined from 27% in 2005 to 17% in 2011. But currently, it has increased to 24% in 2016 and women who are pregnant or breastfeeding are more likely to be anaemic (29% for both groups) as well; these data suggest that anaemia is a public health problem in our country. Increases were observed from 2011 to 2016 in all anaemia categories (6).

The adverse consequences of maternal anaemia may affect not only the neonate and infant but also increase the risk of noncommunicable diseases when the child grows into an adult and the risk of low birth weight in the next generation (7). Maternal anaemia resulted increase in birth weight of fetuses in anaemic mothers (2376.25), compared to nonanaemic mothers (2595 gm) (8).

Morphological changes of placenta due to anaemia condition influences placenta's exchange and haemodynamic processes (9). Although placenta has a remarkable reserve capacity to withstand noxious environment, it is equally true that some unfavorable changes due to maternal anaemia causes adverse effects on placenta which ultimately might compromise the well-being of the fetus (10). As mothers are affected by anaemia, syncytiotrophoblast of entrapped villi degenerates and the stroma of the villi become markedly fibrotic. With this, the villous population decreases further, and the nutritional demands of the fetus are not adequately fulfilled resulting in placental insufficiency and low birth weight babies (11).

Anaemia causes perivillous fibrin depositions which reduces the transfer of essential nutrients from mother to fetus resulted in chronic placental insufficiency; causing low birth weight fetus (8).

Mal-development of placenta is the leading cause of maternal and perinatal mortality and an important factor of fetal growth retardation. Therefore, there is a need to explore the extent of structural changes of placenta, since low haemoglobin level is likely to be related to insufficient functioning of the placenta (12, 13).

## Methods

A comparative cross-sectional study design was employed in Dessie Referral Hospital from May-June, 2018. Dessie town is Northeast of 401 Km from Addis Ababa the capital of Ethiopia. The town is also 478 km far from Bahirdar which is the capital of Amhara regional state. Dessie Referral Hospital is found in Dessie town serving 2.4 million peoples including neighboring zones. It has more than six wards including the Obstetrics and Gynecology ward and the hospitals monthly delivery report is above 500 mothers.

**Study population**: Term anaemic and nonanaemic mothers who attended their delivery in Dessie Referral Hospital during data collection period

**Inclusion criteria**

**Group I**: Anaemic pregnant mothers attend their delivery in DRH during data collection period aged 20–35 years, and diagnosed clinically and haematologically.

**Group II**: Non-anaemic pregnant mothers aged 20–35 years having no signs and symptoms of anaemia with their haemoglobin level recorded to be more than or equal to 11g/dl at any time during pregnancy.

**Exclusion criteria**: Any pathological conditions which affect the placenta as well as the fetus, such as; gestational hypertension, chronic hypertension, and pre-existing diabetes mellitus.

**Sample Size and Sampling procedure**: Sample size was calculated by using Open Epi, version 3.0, taking the difference of means formula, by considering two-sided 95% CI and 80% power. The final calculated sample size was 33 cases, and 33 controls.

**Sampling technique and procedure**: Purposive sampling technique was employed to conduct this study; during data collection period, the number of mothers who were delivered in Dessie Referral Hospital was 550. From these 550 mothers delivered in the hospital, the sample was taken purposively till the total sample size was achieved.

**Data collection tool and procedure**: For each case, preliminary history was elicited from the mother and her clinical sheets regarding current and past medical, surgical, obstetrics and gynecologic histories that affect the microarchitecture of placenta. Hemocque and automated haematology analyzer machine were used to estimate the level of haemoglobin and repeated analysis was also conducted to get rid of some uncertainties. After identifying the cases and controls, we used the World Health Organization's criteria to classify the level of anaemia for this study; mild (10–10.9g/dl), moderate (7–9.9g/dl), and severe (<7g/dl). Then, the fresh placenta was collected as soon as delivery happened and checked for its completeness. The membranes and cord were trimmed off from the placenta in all cases. Then, it was washed by running tap water, cleaned up by towel and labeled with code numbers. After doing this, representative specimens from each fresh placental tissue for microscopy were taken, from margins (peripheral area), center of the placenta, between center and margin, and pathologic area (if any).

Then, after taking the tissue, it was labeled and kept in 10% buffered formalin for fixation. Each tissue was again cut in small pieces of 5 mm × 2mm. Finally, all samples were fixed in 10% buffered formalin solution. After 24 hours embedded in paraffin, 3 histological sections were sectioned using rotatory microtome (3 µm, 4 µm, and 6 µm) on one specimen for representativeness of the tissue sample. All samples were stained with Haematoxylin and Eosin. The biopsy specimens were examined by routine light microscopy using different magnification. Maximum possible fields were examined for each slide to assess: Abnormalities of blood vessels within the villi (Increase in the number of terminal villi vessels, when >6 vessels per terminal villi), Abnormalities of intervillous space such as: Intervillous thrombosis, and width of intervillous space (measured in 5 random fields per slide under 100X objective lens using ocular micrometer and finally mean was calculated), placental calcification, cytotrophoblast proliferation and presence of syncytial knots , focal (>30% of 1 full-thickness slide) and diffuse (≥2 full-thickness slide).

**Data quality assurance**: For ensuring data quality, training was given for data collectors and supervisors concerning on how they take placental tissue, fixation and appropriate disposal of the placenta. Data was collected and recorded using the checklist by 2 BSc midwifery and 1 Medical laboratory staff members working in the same hospital. Finally, the pathologist evaluated the processed tissue blindly.

**Data processing and analysis**: Data were coded, and entered using EPI-data Version 4.2.0 and exported to SPSS Version 22 for analysis. Descriptive statistics like frequency, mean, and standard deviation were computed to describe the study variable and the results were presented by tables and graphs. Association of dependent and independent variables were confirmed by chi-square test. Comparison of intervillous space width in anaemic and non-anaemic placenta was done using one-way ANOVA. Differences p<0.05 was considered statistically significant.

**Ethical Considerations**: Ethical clearance for the beginning of the study was obtained from the Research and Ethics Committee of the Department of Anatomy, School of Medicine, College of Health Sciences, Addis Ababa University. Following approval by the committee, it was submitted to the Institutional Review Board (IRB). After this, letter of cooperation was written by the Department of Anatomy to Dessie Referral Hospital. Every study participant was adequately informed about the objective, benefits and risk of the study. Individual verbal informed consent was obtained from the participants, and those who agreed were included in the study. Then, giving due respect and confidentiality, appropriate disposal of placenta was observed/done by the data collectors and the supervisors.

## Results

**Socio-demographic variables**: The mean maternal age in the study was 27±3.2 years, and 64(66.6%) of the mothers' age was within 25–29 years. The mean (±SD) gestational ages of mothers were 38.4±0.4 and 39.1±1 for anaemic and non-anaemic mothers respectively. Regarding their mode of delivery, 50(76%) of mothers delivered spontaneously by vaginal delivery, and 16(24%) mothers delivered by cesarean section. Out of 66 deliveries, 36(54.5%) of babies were males and 30(45.5%) of them were females. Nearly forty-nine percent of the mothers were multipara.

**Results on placental histopathology**: In this study, 72.5% of the anaemic terminal villi vessels were dilated, compared to 24.2% in non-anaemic placenta. Terminal villi vessels dilation increased as severity of anaemia increased. Based on the result of this study, 75.7% of the anaemic placenta had increase in number of terminal villi vessels, but in non-anaemic placenta, vessel proliferation accounted for 15.15%. Intervillous thrombosis was higher in anaemic placenta compared to noting was observed in non-anaemic placenta (Table 1).

**Table 1:** Distribution of microscopic findings in anaemic and non- anaemic mothers' placenta in Northeast Ethiopia

Microscopic findings	Anaemic (n=33)		Non- anaemic (n=33)	

	Count	Percent	Count	Percent
Vascular dilation	24	72.7 %	8	24.2%
Vascular proliferation	25	75.7%	5	15.1%
Calcification	24	72.7%	18	54.5%
Intervilous thrombosis	17	51.5%	0	0%
Cytotrphoblast proliferation	16	48.5%	1	3.0%
Syncitial knots	27	81.8%	5	15.1%

In the current study, vascular proliferation in the terminal villi of anaemic placenta was prominent, and it was significantly higher in anaemic than non-anaemic (p<0.001). Even though placental calcification was prominent in anaemic than non-anaemic placenta, it was not significantly higher than non-anaemic (p=0.12). Most of anaemic placenta had diffuse syncytial knots, and diffuse syncytial knot significantly increased as severity of anaemia (p<0.001) (Table 2).

**Table 2:** Chi-square test between anaemic and non-anaemic groups with respect to histopathologic findings

Microscopic findings	Non-anaemic (n=33)	Anaemic (n=33)	χ^2^- statistic
Vascular dilation	8	24	15.52 *
Vascular proliferation	5	25	24.44 *
Calcification	18	24	2.35
Intervillous thrombosis	0	17	22.89 *
Cytotrphoblast proliferation	1	16	17.82*
Syncitial knots	5	27	29.02*

* Significant

In this study, intervillous space was wider in anaemic placenta compared to non-anaemic placenta. There was a significant difference in intervillous width of anaemic and non-anaemic groups (ANOVA= 64.03, p< 0.001) (Table 3).

**Table 3:** One-way ANOVA shows width of intervillous space in anaemic and non- anaemic mothers' placenta in Northeast Ethiopia

Maternal status	Mean ± SD(µm)	ANOVA	p-value
Non-anaemic	19.3±1.8	64.03	< 0.001
Mild anaemic	27.5±6.4		
Moderate anaemic	33.0±5.5		
Severe anaemic	40.3±3.4		

ANOVA- Analysis of Variance

* µm- Micrometer

## Discussion

Histopathological studies of placenta under different magnifications revealed variations from one placenta to the other. In the current study, structure of intervilous space, chorionic villi parts and vessels were varied in anaemic and non-anaemic group of mothers. In the placenta of maternal anaemia cases, it was observed that 72.7% of terminal villi capillaries were dilated compared to 24.2% of terminal villi capillaries dilation in non-anaemic placentas. Dilation of terminal villi vessels significantly increased with severity of anaemia (p=0.001). This result was in line with the study conducted by Soni and Nair in which anaemic placental capillaries per villi were seen to be increased in number and dilated with severity of anaemia(14). This may be due to compensatory response for hypoxia as a result of maternal anaemia.

In the current study, increase in number of capillaries per villi in anaemic placenta was 75.7%, compared to 15.1% in non-anaemic placenta. Capillary number per villi was significantly increased with severity of anaemia (p <0.001). This finding was in line with other studies conducted by Lelic et al., in which hypoxia as a consequence of maternal anaemia, lead to significant increase of terminal villi blood vessels(14,15). This may be due to hypoxic placenta as a result of maternal anaemia increase in terminal villi vessels as a compensatory response.

Regarding intervillous space width of placenta, intervillous space of anaemic placenta was wider than non-anaemic placenta. There was a statistically significant difference between groups (ANOVA= 64.03, p<0.001). This finding was comparable with the study conducted in Pakistan in which width of intervillous space in anaemic placenta was wider, compared to non-anaemic placenta and there were a statistically significant difference between groups (p<0.05)(16). This finding was also parallel to the study conducted in India, in which 60% of anaemic placenta had wider intervillous space compared to 6.6% in non-anaemic placenta (10). The increment in width of intervillous space in anaemic placenta might be the reduction in number of villi with a consequential increase in intervillous space.

In this study, 51.5% of anaemic placenta had intervillous thrombosis, compared to nothing was observed in non-anaemic placenta, and space thrombosis significantly increased with severity of anaemia (p<0.001). This finding was not comparable with other study conducted in India, in which 50% of anaemic placenta had intervillous thrombosis, compared to 30% intervillous thrombosis in non-anaemic placenta (10). This may be due to the discrepancy in inclusion and exclusion criteria of study participants. The supremacy of intervillous thrombosis in anaemic placentas may be due to mixing of fetal and maternal blood in the space of villi resulted from rupture of villus at the site of syncytial thinning.

Cytotrophoblast proliferation in anaemic placenta was 48.5%, compared to non-anaemic placenta which accounted for 3%. This result was supported by Baske, in which 30% of placentas in anaemic mothers developed cytotrophoblast proliferation, compared to 13% in non-anaemic groups(10).

Another study revealed that cytotrophoblastic proliferation was not found in any of non-anaemic placenta, whereas in anaemic placenta, cytotrophoblastic proliferation was increased as severity of anaemia increases (14). This may be due to the fact that the tissues undergo ischemic damage as a result of low oxygen tension, in maternal anaemia.

In this study, even though syncytial knot was evident in non-anaemic placentas, the degree of knot formation was significantly increased in anaemic placentas (p<0.001). For instance, 81.8% of anaemic placenta were developed diffuse syncytial knots, whereas, in non-anaemic placenta, 15.1% was diffuse knots. This result was in line with a study conducted in India, in which incidence of syncytial knot formation in anaemic group was 83% compared to 10% in non-anaemic group. The possible reason behind the increase in diffuse syncytial knots in maternal anaemia may be due to the effort being made to form new villi as a compensatory response to increase an effective surface area for exchange of substances (10,14). In conclusion, chorionic villi capillaries increased in number, and it was dilated in anaemic placenta, compared to non-anaemic. Intervillous space and thrombosis were significantly increased in anaemic placenta. Diffuse syncytial knot was significantly increased in anaemic compared to non-anaemic placenta.

This study was limited only on histopathology of placenta, for instance, it cannot proceed to immunohistochemistry. The study used small number of participants; hence, difficulty of generalization.

Clinicians should carry out routine placental gross and microscopic examination during postpartum period; hence, this will provide better evidence for clinical decisions. On time histopathologic placental, interpretation should also be carried out on sick mothers and babies before further referral. Large scale study should be conducted regarding the effects of anaemia on placental histopathology including histochemistry using the current study as a baseline data.

## Figures and Tables

**Figure 1:**
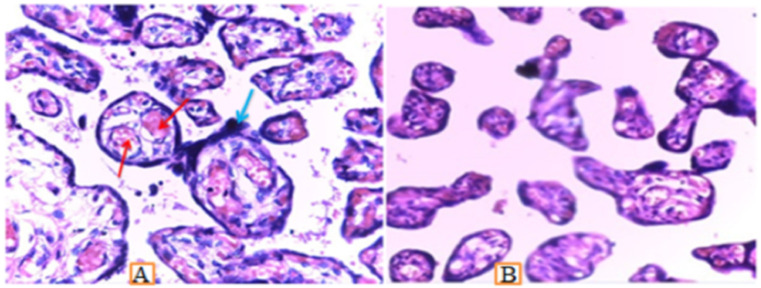
Photomicrograph showing markedly increased dilated and congested terminal villi vessels (red arrows) and syncytial knots (blue arrows) in anaemic placenta (A) compared to non-anaemic placenta (B), Stain used Hematoxyline and Eosin, 60X.

**Figure 2:**
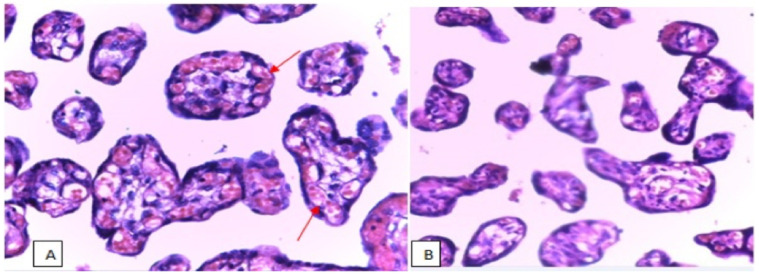
Photomicrograph showing increase in number of capillaries (proliferation) in the terminal villi of placenta (arrows), in anaemic placenta (A) than non-anaemic placenta (B) in Northeast Ethiopia, Stain used Hematoxyline and Eosin, 40X.
